# Factors influencing breast cancer screening among reproductive age women in Lesotho: Analysis of the 2023-24 demographic and health survey using the Andersen Behavioral Model

**DOI:** 10.1371/journal.pone.0336983

**Published:** 2025-11-13

**Authors:** Mesfin Abebe, Yordanos Sisay Asgedom, Amanuel Yosef Gebrekidan, Tsion Mulat Tebeje

**Affiliations:** 1 Department of Midwifery, College of Health Sciences and Medicine, Dilla University, Dilla, Ethiopia; 2 Department of Epidemiology and Biostatistics, College of Health Sciences and Medicine, Wolaita Sodo University, Wolaita Sodo, Ethiopia; 3 School of Nursing and Midwifery, Edith Cowan University, Joondalup, Western Australia, Australia; 4 School of Public Health, College of Health Sciences and Medicine, Wolaita Sodo University, Wolaita Sodo, Ethiopia; 5 School of Public Health, College of Health Sciences and Medicine, Dilla University, Dilla, Ethiopia; University of Adelaide School of Medical Sciences: The University of Adelaide Adelaide Medical School, AUSTRALIA

## Abstract

**Background:**

Breast cancer is the leading cause of cancer-related deaths in women globally and a significant public health burden in sub-Saharan Africa, which accounts for approximately 15% of all cancer-related mortality. In sub-Saharan Africa, breast cancer incidences increased by 247% from 1990 to 2019. In Lesotho, breast cancer is the second most common cancer affecting women, a situation worsened by a fragile healthcare system and low screening rates. Despite its high morbidity and mortality, there is limited understanding of the factors influencing breast cancer screening among women of reproductive age. This study aims to identify these factors by utilizing the newly released Lesotho DHS dataset and the Anderson Behavioral Model.

**Methods:**

This study utilized a cross-sectional design with data from the recent Lesotho Demographic and Health Survey (LDHS), which employed a stratified two-stage sampling method across 400 Enumeration Areas and 9,976 households. This analysis included a weighted sample of 6,413 reproductive-age women (15–49 years) to determine factors of breast cancer screening. The predisposing, enabling, and need factors were examined using the Andersen Behavioral Model. Stata version 16 was used for a multilevel mixed-effects logistic regression model. Results were presented as adjusted odds ratios (AOR) with 95% confidence intervals, and a P-value less than 0.05 was considered statistically significant.

**Results:**

The prevalence of breast cancer screening among women of reproductive age in Lesotho was 22.20% (95% CI 21.19–23.23). Significant factors included age 25–34 (AOR = 1.54; 95% CI 1.26–1.88), age 35–49 (AOR = 2.10; 95% CI 1.71–2.58), healthcare facility visits in the past 12 months (AOR = 1.47; 95% CI 1.26–1.71), health insurance coverage (AOR = 1.86; 95% CI 1.36–2.53), high media exposure (AOR = 1.23; 95% CI 1.01–2.52), contraceptive use (AOR = 1.18; 95% CI 1.03–1.37), and parity: multiparous (AOR = 2.29; 95% CI 1.84–2.85) and grand multiparous (AOR = 1.67; 95% CI 1.16–2.40).

**Conclusion:**

This finding that 22.2% of reproductive age women in Lesotho underwent breast cancer screening highlights a pressing gap in preventive health efforts. The Andersen Behavioral Model underscores key determinants that significantly influence breast cancer screening uptake in our study. Significant factors included age, healthcare facility visits, health insurance coverage, media exposure, contraceptive usage, and parity. These findings underscore the need for targeted interventions that address model-based determinants to improve breast cancer screening uptake.

## Introduction

Breast cancer remains a significant health concern worldwide and ranks as one of the leading causes of cancer mortality among women, particularly in low- and middle-income countries [1,2]. Despite the rising incidence of breast cancer, early detection through screening has been largely suboptimal, resulting in late-stage diagnoses and poor outcomes for many affected women [3]. Globally, an estimated 2.3 million women were diagnosed with breast cancer in 2020, leading to approximately 685,000 deaths, with many of these occurring in sub-Saharan Africa where awareness and screening practices are critically low [3,4]. By 2050, new cases and deaths are projected to rise by 38% and 68%, respectively, with low HDI countries being disproportionately affected [5]. This situation underscores the significant challenges faced by low- and middle-income countries (LMICs), driven by disparities in healthcare access and outcomes [6].

Breast cancer is the leading cause of cancer-related deaths in women globally and a significant public health burden in sub-Saharan Africa, which accounts for approximately 15% of all cancer-related mortality [7]. In sub-Saharan Africa, breast, cervical, and prostate cancers dominate the overall cancer burden, with breast cancer accounting for 25% of new cases in women [8,9]. By 2040, breast cancer is expected to result in over 3 million new cases and 1 million deaths annually due to population growth and aging alone [10]. In Africa, breast cancer accounts for 25% of all diagnosed cancers and is responsible for 20% of cancer-related deaths among women. The incidence of breast cancer in Africa is steadily rising and is expected to double by 2050 [11]. In sub-Saharan Africa, breast cancer incidences increased by 247% from 1990 to 2019 [8]. Despite improved survival rates, significant disparities exist between countries due to factors like inadequate early-stage screening, detection, and affordable therapy [12].

Although breast cancer mortality is higher in Africa than in high-income countries, the disease has not been extensively studied in the region [13]. Moreover, the burden of breast cancer in women aged 15–49 is particularly concerning, as this age group is less studied and their health impacts extend to their families and society [1]. Breast cancer is a major global public health issue, especially in low-resource countries. Screening services are effective for early detection [14]. In low-resource settings, the WHO recommends clinical breast examination (CBE) as the most cost-effective screening for women [15]. The study found that breast cancer screening utilization in 14 low-resource countries was 15.41%, with variations of 81.10% in Europe, 18.61% in Asia, 14.30% in the Americans, and 14.29% in Africa [14]. A survey in four sub-Saharan African countries revealed a 12.9% prevalence of breast cancer screening [16]. Another study showed that 18.4% of women in Ghana utilized breast cancer screening services [17]. A study in Tanzania found a 5.2% prevalence of breast cancer screening [18]. Factors associated with screening included age, educational status, residence, marital status, health insurance coverage, wealth index, distance to health facilities, parity, contraceptive use, media exposure, and breast cancer awareness [16,17,19–21]. According to the 2014 DHS, breast cancer screening utilization in Lesotho was 9.73% [3]. So, the utilization of breast cancer screening services is low in many low-resource countries, including Lesotho [3,14]. While breast cancer cannot be entirely prevented, adopting lifestyle changes and early diagnosis significantly reducing incidence and mortality rates. Therefore, screening is crucial for early breast cancer detection and significantly reduces mortality rates [22].

In Lesotho, breast cancer is the second most common cancer among women, with a weak healthcare system and low screening rates [20]. Together, breast and cervical cancers account for approximately 43% of all annual cancer diagnoses in the country [23]. The 2023−24 Lesotho Demographic and Health Survey (DHS) offers a unique opportunity to identify the prevalence and determinants of breast cancer screening in this population. Despite the high mortality rates associated with breast cancer, the prevalence of screening among women of reproductive age in Lesotho remains alarmingly low [20]. Previous research in sub-Saharan Africa has highlighted several key determinants, including education level, age, health insurance coverage, and socioeconomic status [16,24]. Access to healthcare services and health insurance coverage, along with socio-demographic and economic factors, significantly correlate with higher breast cancer screening utilization. Additionally, screening behaviors, prior knowledge, and physician access influence women’s participation in screening services [14].

However, a significant gap exists in the literature regarding the specific context of Lesotho. In this country, breast cancer has become one of the leading causes of morbidity and mortality among women. Despite this, there is a limited understanding of the factors that influence breast cancer screening services for women of reproductive age [20]. Most of the existing studies in Lesotho have focused on factors associated with awareness of breast cancer screening, primarily utilizing data from the 2014 Demographic and Health Survey (DHS) dataset [3,20,25,26]. However, this dataset is now outdated. Updated information based on the newly released Lesotho DHS dataset, analyzed through the lens of the Anderson Behavioral Model, could be instrumental in identifying the factors associated with the utilization of breast cancer screening among women of reproductive age. This study aims to explore these determinants using the Andersen Behavioral Model, which provides a comprehensive framework for understanding health service utilization [24]. The Andersen Behavioral Model was chosen for its relevance in explaining health service utilization, particularly in preventive care such as cancer screening. This model conceptualizes service use as being influenced by three domains: predisposing factors (such as socio demographic traits), enabling factors (including access and resources), and need factors (referring to perceived or actual health status) [27]. It has been extensively applied in studies throughout sub-Saharan Africa and globally to investigate disparities in screening uptake [28–30]. This updated analysis would provide valuable insights for policymakers, enabling them to develop more effective strategies to improve breast cancer screening rates and ultimately enhance women’s health outcomes in Lesotho.

## Methods

### Study design and data source

This study utilized a cross-sectional design, drawing data from the 2023−24 Lesotho Demographic and Health Survey (LDHS). The LDHS is a nationally representative survey that collects data on a wide range of health and demographic indicators. The survey employs a stratified two-stage sampling design to ensure representativeness across different regions and population groups in Lesotho. In the 2023/24 Lesotho Demographic and Health Survey (DHS), the sampling process began by identifying 400 Enumeration Areas (EAs) as clusters. Households within these clusters were then selected randomly, employing the Probability Proportion to Size (PPS) method to ensure a representative distribution. From each cluster, 25 households were systematically chosen, resulting in a total of 9976 households being included in the survey. Given that the study focused on reproductive-age women, we utilized the individual (women’s) Record dataset (IR file). For this analysis, 6413 women of reproductive age were included.

### Study population

The study population comprised women of reproductive age (15–49 years) residing in Lesotho. Inclusion criteria were women who had complete data on breast cancer screening and relevant covariates. Women with missing or incomplete data on key variables were excluded from the study.

### Outcome variable

The primary outcome variable was whether women had their breasts examined for cancer by a healthcare provider. This could involve a clinical breast exam, where hands are used to feel for lumps or other changes, or imaging techniques like mammograms. The specific survey question used to assess this was: “Has a doctor or other healthcare provider examined your breasts to check for breast cancer?” Responses were dichotomized as “yes” or “no,” allowing for a binary logistic regression framework for analysis.

### Theoretical framework

The Andersen Behavioral Model was used as the theoretical framework to guide the selection and analysis of variables [27]. This model posits that healthcare utilization is influenced by predisposing factors (e.g., age, education), enabling factors (e.g., health insurance, access to healthcare), and need factors (e.g., perceived health status, knowledge of disease) [28].

### Independent variables

The selection of study variables was guided by a comprehensive review of existing literature, ensuring that the variables chosen were both relevant and supported by previous research findings. Additionally, the availability of these variables within the dataset was a crucial consideration, ensuring that the data required for analysis was accessible and complete [16,17,20,21,31]. The Andersen Behavioral Model served as the theoretical framework for this study, providing a structured approach to categorize and analyze the determinants of breast cancer screening. This model emphasizes the importance of predisposing, enabling, and need factors in understanding health service utilization, and thus, variables were selected to align with these categories. The independent variables included both individual-level and community-level factors, classified according to the Andersen Behavioral Model [27].

Predisposing Factors**:** These include women’s age (categorized as 15–24, 25–34, and 35–49 years), educational level (no formal education, primary, secondary, and higher), parity (nulliparous, multiparous, and Grand multiparous), marital status (unmarried, or married), sex of household head (male or female) and literacy (literate or illiterate). **Enabling Factors**: This category encompasses economic status, determined by wealth index (poorest, poorer, middle, richer, and richest); residence (urban or rural); working status (yes or no), media exposure (no media exposure, low media exposure, and high media exposure), internet use (yes or no), distance to health facility (big problem or not big problem), contraceptive user (yes or no), and health insurance coverage (yes or no). **Need Factors:** These factors encompass health perceptions and awareness related to breast cancer. Variables include self-reported health status (categorized as good, moderate, or poor) and healthcare facility visits in the last 12 months (yes or no).

### Operational definitions

Media exposure: The media exposure score was calculated using the frequency of reading newspapers, listening to the radio, and watching television. Each variable was coded as 0 (not at all), 1 (less than once a week), and 2 (at least once a week). The total score ranged from 0 to 6 and was categorized as no exposure (0), low exposure (1–3), and high exposure (4–6) [32].

Self-reported health status: Self-reported health status was assessed with the question, ‘In general, how would you rate your health?’ on a Likert scale from very good to very bad. Responses were categorized as good (very good and good), moderate, and poor (bad and very bad) [33–35].

### Data processing and statistical analysis

Before conducting any statistical analysis, the data were weighted using sampling weights, primary sampling units, and strata to ensure valid and representative estimates. Data were analyzed using Stata version 16. Descriptive statistics were used to summarize the characteristics of the study population. Bivariate analyses were conducted to examine the associations between independent variables and breast cancer screening uptake. A multilevel mixed-effects logistic regression model was employed to account for the hierarchical structure of the DHS data, where women are nested within communities or geographic regions. To identify the best-fitting model, four hierarchical models were constructed: a null model with no explanatory variables (Model I), a model with individual-level variables (Model II), a model with community-level variables (Model III), and a full model combining both individual and community level variables (Model IV). Model comparison was based on deviance (−2LL) and log-likelihood ratio, with the model having the lowest deviance (−2LL) and the highest log-likelihood ratio indicating the best fit [36]. In addition, the random variability in breast cancer screening uptake was assessed using the intra-class correlation coefficient (ICC), proportional change in variance (PCV), and median odds ratio (MOR).

The initial model was a null model containing no explanatory variables to estimate the intra-class correlation coefficient (ICC), which indicates the proportion of total variability attributable to grouping. Subsequent models included individual-level predictors, community-level predictors, and a combined model with both levels. Variables with p-values less than 0.1 from the bivariate analysis were included in the multivariable model. The best model was selected during construction using post-estimation methods, including log-likelihood, PCV, and deviance (−2 log-likelihood). To assess multicollinearity, variance inflation factors (VIF) were calculated for the variables in the models, resulting in a mean VIF of 1.31 for the final model. The significance of variables was assessed using adjusted odds ratios (AORs) with corresponding 95% confidence intervals (CI). Variables with p-values less than 0.05 were considered statistically significant.

### Ethical considerations

Because this study used secondary data from the Demographic and Health Surveys (DHS), ethical approval or participant consent was not necessary. We received permission to download and use the data from the DHS Program for this study. The datasets do not include any personal identifiers such as names or addresses of individuals or households. Confidentiality and anonymity of the respondents were maintained throughout the study.

## Results

A total of 6413 weighted reproductive-age women were included in this study. Among the respondents, 36.78% of women were in the age group 15–24 years, and 57.41% had secondary-level education. Additionally, 50.35% of the reproductive-age women were currently unmarried. More than half of the participants (54.50%) lived in rural areas, and 56.96% of the households were headed by males. More than two-thirds (67.38%) of participants visited healthcare facilities in the last 12 months, and more than three-fourths (75.86%) of participants did not consider distance to a healthcare facility a big problem. A significant portion of participants did not have health insurance (95.78%). Nearly half (49.80%) of reproductive-age women reported low media exposure, while a large majority used the internet (82.59%). Additionally, 56.28% of participants rated their self-reported health status as good (Table 1).

**Table 1:** Descriptive characteristics of reproductive-age women in Lesotho.

Variables	Category	Weighted frequency (%)	Breast cancer screening	
Yes (%)	No (%)
Age	15-24 years	2359(36.78)	228(9.68)	2131(90.32)
	25-34 years	1766(27.55)	466(26.36)	1300(73.64)
	35-49 years	2288(35.67)	729(31.89)	1559(68.11)
Education level	No education	39(0.60)	6(15.38)	33(84.62)
	Primary	1595(24.88)	341(21.39)	1254(78.61)
	Secondary	3682(57.41)	756(20.53)	2926(79.47)
	Higher	1097(17.10)	320(29.19)	777(70.81)
Marital status	Not married	3229(50.35)	589(18.25)	2640(81.75)
	Married	3184(49.65)	834(26.20)	2350(73.80)
Residence	Urban	2918(45.50)	727(24.91)	2191(75.09)
	Rural	3495(54.50)	696(19.93)	2799(80.07)
Sex of household head	Male	3653(56.96)	798(21.86)	2855(78.14)
	Female	2760(43.04)	625(22.65)	2135(77.35)
Healthcare facility visits in the last 12 months	No	2092(32.62)	330(15.80)	1762(84.20)
	Yes	4321(67.38)	1093(25.29)	3228(74.71)
Distance to a healthcare facility	Big problem	1548(24.14)	290(18.73)	1258(81.26)
	Not a big problem	4865(75.86)	1133(23.29)	3732(76.71)
Health insurance	No	6143(95.78)	1318(21.46)	4825(78.54)
	Yes	270(4.22)	105(38.95)	165(61.05)
Media exposure	No media exposure	1321(20.60)	232(17.56)	1089(82.44)
	Low media exposure	3194(49.80)	675(21.14)	2519(78.86)
	High media exposure	1898(29.60)	516(27.20)	1382(72.80)
Use of Internet	No	1116(17.41)	215(19.27)	901(80.73)
	Yes	5297(82.59)	1208(22.81)	4089(77.19)
Literacy level	Illiterate	99(1.55)	13(13.61)	86(86.39)
	Literate	6314(98.45)	1410(22.33)	4904(77.67)
Wealth index	Poorest	894(13.94)	137(15.32)	757(84.68)
	Poorer	1055(16.45)	178(16.87)	877(83.13)
	Middle	1253(19.54)	288(22.98)	965(77.02)
	Richer	1564(24.39)	382(24.42)	1182(75.58)
	Richest	1647(25.68)	439(26.65)	1208(73.34)
Currently Working	No	3848(60.01)	651(16.92)	3197(83.08)
	Yes	2565(39.99)	772(30.10)	1793(69.90)
Contraceptive use history	No	2892(45.09)	510(17.63)	2382(82.37)
	Yes	3521(54.91)	913(25.93)	2608(74.07)
Parity	Nulliparous	2032(31.69)	199(9.80)	1833(90.20)
	Multiparous	4079(63.61)	1160(28.44)	2919(71.56)
	Grand multiparous	302(4.70)	64(21.32)	237(78.68)
Self-reported health status	Poor	568(8.85)	158(27.90)	410(72.10)
	Moderate	2236(34.87)	506(22.63)	1730(77.37)
	Good	3609(56.28)	759(21.03)	2850(78.97)

### Prevalence of breast cancer screening among reproductive-age women in Lesotho

**Fig 1:**
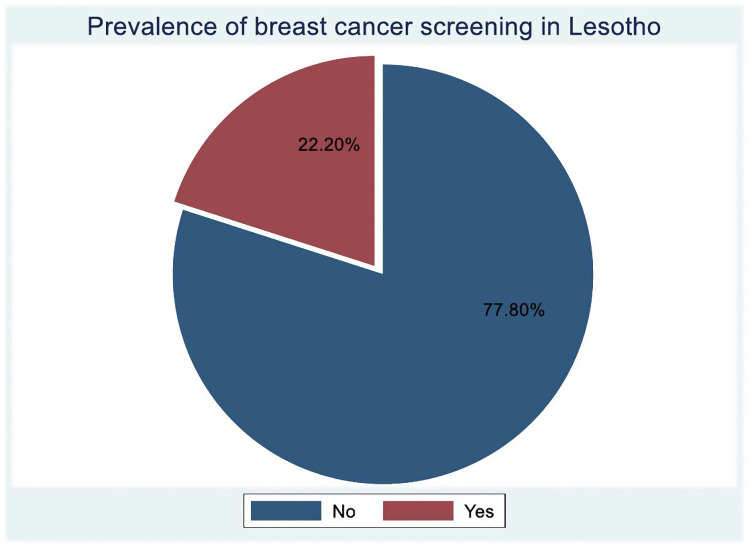
Prevalence of clinical breast cancer screening among reproductive-age women in Lesotho.

The prevalence of breast cancer screening among women of reproductive age in Lesotho was 22.20% (95% CI 21.19–23.23) (Fig 1).

### Random effect and model fitness

The model fitness statistics assess the fit of the multilevel logistic regression model to the data. The ICC in the null model was 0.04, indicating that 4% of the variation in breast cancer screening is due to community-level factors (between-cluster variability). The highest PCV value in the final model suggests that most of the variation in breast cancer screening is explained by individual and community-level factors. Model IV had the highest log-likelihood, making it the most suitable model. Additionally, the lowest deviance value in the final model indicates that Model IV is the best explanatory model for the variation in breast cancer screening between clusters (Table 2).

**Table 2:** Factors influencing clinical breast cancer screening among reproductive-age women in Lesotho.

Variables	Null model	Model II AOR (95%CI)	Model III AOR (95%CI)	Model IV AOR (95%CI)
**Age**				
15-24 years		1		**1**
25-34 years		1.54(1.27, 1.88)		1.54(1.26, 1.88) *
35-49 years		2.10(1.71, 2.58)		2.10(1.71, 2.58) *
**Education level**				
No education		1		1
Primary		0.63(0.30, 1.33)		0.63(0.30, 1.34)
Secondary		0.78(0.36, 1.66)		0.77(0.36, 1.64)
Higher		0.96(0.44, 2.09)		0.93(0.43, 2.03)
**Marital status**				
Not married		**1**		**1**
Married		0.96(0.82, 1.12)		0.96(0.82, 1.13)
**Sex of household head**				
Male		0.89(0.77, 1.03)		0.90(0.78, 1.04)
Female		1		**1**
**Parity**				
Nullparous		1		**1**
Multiparous		2.28(1.83, 2.84)		2.29(1.84, 2.85) *
Grand multiparous		1.63(1.13, 2.35)		1.67(1.16, 2.40) *
**Health insurance**				
No		1		1
Yes		1.87(1.37, 2.56)		1.86(1.36, 2.53) *
**Media exposure**				
No media exposure		1		**1**
Low media exposure		1.20(1.02, 1.42)		1.17(0.98, 1.38)
High media exposure		1.29(1.05, 1.59)		1.23(1.01, 1.52) *
**Use of Internet**				
No		1		**1**
Yes		0.96(0.80, 1.15)		0.96(0.80, 1.15)
**Literacy level**				
Illiterate		1		1
Literate		1.23(0.71, 2.13)		1.24(0.72, 2.16)
**Currently working**				
No		1		1
Yes		1.15(0.99, 1.33)		1.13(0.97, 1.31)
**Contraceptive use**				
No		1		1
Yes		1.17(1.01, 1.35)		1.17(1.02, 1.35) *
**Healthcare facility visits in the last 12 months**				
No		1		**1**
Yes		1.47(1.26, 1.71)		1.47(1.26, 1.71) *
**Self-reported health status**				
Poor		1		**1**
Moderate		1.04(0.83, 1.32)		1.03(0.82, 1.30)
Good		1.04(0.83, 1.31)		1.03(0.82, 1.30)
**Distance to a healthcare facility**				
Big problem			1	1
Not a big problem			1.17(1.01, 1.37)	1.14(0.97, 1.34)
**Residence**				
Urban			1	1
Rural			0.78(0.67, 0.90)	0.91(0.77, 1.07)
**Model Comparison and Fitness**				
Community level variance	0.14	0.094	0.11	0.093
PCV	Reference	32.85%	21.42%	33.57%
LR test	X^2^ = 20.07, P, < 0.0001			
ICC% (95%CI)	4.0	2.7	3.4	2.6
Log-likelihood	**−**3202.3711	−2982.0666	−3192.5678	−2979.5425
Deviance	6404.7422	5964.1332	6385.1356	5959.085

*P- value <0.05.

### Factors associated with breast cancer screening in Lesotho

In the final model (model IV) of multivariable multilevel logistic regression, several factors were significantly associated with breast cancer screening among reproductive-age women in Lesotho. These factors included age, healthcare facility visits within the past 12 months, health insurance coverage, media exposure, contraceptive usage, and parity.

Participants aged 25–34 years were 54% more likely to be screened for breast cancer compared with younger participants aged 15–24 years (AOR = 1.54; 95%CI (1.26, 1.88). Similarly, women aged 35–49 years were 2.10 times more likely to undergo breast cancer screening compared to women aged 15–24 years (AOR = 2.10; 95% CI (1.71, 2.58)). Women of reproductive age who visited a healthcare facility in the past 12 months were 47% more likely to undergo breast cancer screening compared to those who did not visit a healthcare facility (AOR = 1.47; 95% CI (1.26, 1.71)). Women of reproductive age with health insurance coverage were 1.86 times more likely to undergo breast cancer screening than those without insurance (AOR = 1.86; 95% CI (1.36, 2.53)). The odds of breast cancer screening among women with high media exposure were 1.23 times greater than those among women with no media exposure (AOR = 1.23; 95% CI (1.01, 2.52)). Furthermore, women who use contraceptives are 1.18 times more likely to undergo breast cancer screening (AOR = 1.18; 95% CI (1.03, 1.37)). Multiparous and grand multiparous women were 2.29 and 1.67 times more likely to undergo breast cancer screening compared to nulliparous women, respectively (AOR = 2.29; 95% CI: 1.84–2.85) and AOR = 1.67; 95% CI: 1.16–2.40)) (Table 2).

## Discussion

This study aimed to assess the prevalence and factors influencing breast cancer screening among women of reproductive age in Lesotho, using Andersen’s healthcare utilization model as a theoretical guide. The model considers predisposing, enabling, and need factors that influence healthcare utilization. The study utilized data from the recent Lesotho Demographic and Health Survey (LDHS) to provide a comprehensive analysis. The findings revealed that the prevalence of breast cancer screening among women of reproductive age in Lesotho was 22.20%. While this figure is higher than in some other sub-Saharan African countries, it still highlights a significant gap in screening coverage. A previous study in Lesotho reported a clinical breast examination (CBE) uptake rate of 9.73% [20]. This lower figure, compared to the current 22.20% prevalence, suggests that although awareness and screening efforts have improved, a substantial portion of the population remains without access to these essential services. Our findings indicated a higher prevalence of clinical breast cancer screening compared to various studies. For instance, a multilevel analysis conducted across six sub-Saharan African countries found an overall prevalence of clinical breast cancer screening was 14.23% [21]. Further studies illustrate varying rates of screening in the region: seven additional sub-Saharan African countries reported a prevalence of 19.2% [37], while specific countries revealed the following figures: Nepal at 6.5% [38], Ghana at 18.39% [39], Kenya at 12% [31], and Tanzania at 6% [40]. Additionally, a multilevel analysis of five sub-Saharan African countries revealed a prevalence of 16.3% [19], while a multi-country analysis of 14 low-resource countries indicated an average screening rate of 15.41% [41]. Interestingly, these figures contrast with the reported prevalence of 22.20% in Lesotho, suggesting that Lesotho exhibits an above-average screening uptake when compared to the overall regional statistics. Another study focusing on breast cancer screening within four sub-Saharan African countries specifically Burkina Faso, Ivory Coast, Kenya, and Namibia found an overall prevalence of 12.9%. Among these countries, Namibia recorded the highest screening rate at 23.1%, while Ivory Coast had the lowest at 5.2% [16]. Thus, the prevalence of 22.20% in Lesotho aligns closely with Namibia’s, indicating that both nations possess relatively higher screening rates within the sub-Saharan context. Additionally, our findings are consistent with those of a systematic review and meta-analysis conducted in low- and middle-income countries, which reported a clinical breast examination screening prevalence of 23.1% [42]. This alignment underscores the potential efficacy of Lesotho’s screening programs and highlights the need for continued efforts to enhance awareness and access to breast cancer screening across the region.

Our study found that the prevalence of clinical breast cancer screening is lower compared to other countries, with Thailand having a screening prevalence of 29% [43], Iran at 60.2% [22], and Vietnam at 51% [44]. These figures suggest that while our findings indicate a lower uptake of breast cancer screening, there are successful models from these countries that might guide future enhancements in screening programs and health education initiatives. The variation in the prevalence of clinical breast cancer screening can be attributed to several factors. In Lesotho, the prevalence is higher than in many sub-Saharan African countries, indicating improved awareness and screening efforts. The use of recent demographic and health survey datasets also highlights these differences. Additionally, disparities in healthcare access, socioeconomic status, and educational levels contribute to the variation in screening rates between countries.

This study applied Andersen’s Behavioral Model to examine the determinants of breast cancer screening utilization, revealing a clear interplay between predisposing, enabling, and need factors [28,45]. In this finding, predisposing characteristics such as age and parity significantly influenced screening behaviour. Older women, particularly those aged 25–34 and 35–49, were more likely to undergo screening compared to their younger counterparts. This finding is consistent with previous studies [16,21,24,26,40], indicating that older women are more aware of the importance of breast cancer screening and are more inclined to participate in such programs. This trend may be due to increased health awareness, more frequent healthcare interactions, and a higher perceived risk of breast cancer among older women. Similarly, our multilevel analysis revealed that being multiparous or grand multiparous women increases breast cancer screening compared to being nulliparous women. This finding aligns with previous studies [19–21,24]. The possible reason for the higher likelihood of breast cancer screening among women with higher parity may be attributed to several factors. Women with higher parity, meaning those who have given birth to multiple children, are more likely to undergo screening due to increased interactions with healthcare providers during pregnancy and childbirth.

Our study findings strongly affirm the role of enabling factors in shaping breast cancer screening behavior, particularly through health insurance coverage, media exposure, and contraceptive use. This study showed that health insurance coverage significantly influences breast cancer screening. Women with health insurance are more likely to undergo screening than their counterparts. This finding aligns with studies [16,19,20,39,40,46], indicating that health insurance increases the likelihood of participating in preventive health services, including breast cancer screening. According to the current study, high media exposure was positively associated with breast cancer screening. Studies have shown that women regularly exposed to health information through media are more likely to participate in screening programs [19–21,24,39,41]. This is because media exposure plays a crucial role in raising awareness about breast cancer and the importance of early detection. The study revealed that contraceptive use is significantly associated with breast cancer screening. This finding aligns with previous studies [19,24,39], showing a positive association between contraceptive use and participation in preventive health services. Women who use contraceptives are more likely to be in regular contact with healthcare providers, increasing their chances of receiving information about breast cancer screening.

According to Andersen’s Behavioral Model, need factors, both perceived and evaluated, are the most immediate determinants of health service utilization [28,45]. In this study, recent visits to healthcare facilities were significantly associated with increased breast cancer screening uptake. This reflects the models assertion that contact with health services often activates perceived health needs and facilitates preventive action. Women who were frequent visitors to healthcare facilities in the past 12 months were positively associated with breast cancer screening. This aligns with other studies [19–21,24,26,39] that emphasize the role of healthcare access in promoting screening. Women who regularly visit these facilities are more likely to receive information about the importance of screening and have improved access to screening services. So, our findings affirm that strengthening routine healthcare access and integrating screening promotion into everyday clinical encounters can significantly improve breast cancer screening rates.

Overall, our findings conclude that Andersen’s model is a strong framework for understanding breast cancer screening behaviour. The patterns we observed highlight a dynamic interplay between individual predisposing factors, enabling factors, and perceived health needs. Future interventions should take these dimensions into account to improve screening coverage, especially among underserved populations.

## Strengths and limitations of the study

This study utilizes a large, nationally representative sample from the recent Demographic and Health Survey, enhancing the generalizability of the findings. The application of the Andersen Behavioral Model offers a comprehensive framework for understanding the determinants of breast cancer screening, which is considered a strength of our study. However, there are limitations, including the cross-sectional design that restricts the ability to infer causality. Additionally, the reliance on self-reported data may introduce recall bias and social desirability bias. Further, longitudinal studies are needed to confirm these findings and explore causal relationships.

## Conclusion and recommendation

The prevalence of breast cancer screening among women of reproductive age in Lesotho was 22.20%. Significant factors associated with clinical breast cancer screening include age, healthcare facility visits, health insurance coverage, media exposure, contraceptive usage, and parity. These findings highlight the need for targeted interventions to improve screening rates, aligning with the Sustainable Development Goals (SDGs), particularly Goal 3 (Good Health and Well-being). By addressing these factors, we can enhance awareness, access, and affordability of breast cancer screening services. The Anderson Behavioral Model highlights the significance of predisposing, enabling, and need factors in healthcare utilization. Our study confirms that age and parity (predisposing factors), health insurance coverage, contraceptive use, and media exposure (enabling factors), along with healthcare facility visits (need factors), significantly influence breast cancer screening behaviour. By understanding these determinants, policymakers and healthcare providers can develop effective strategies to increase screening rates and ensure equitable access to preventive health services. Policymakers and healthcare providers should focus on increasing access to healthcare, promoting health insurance coverage, leveraging media for awareness campaigns, and encouraging regular healthcare visits to improve breast cancer screening uptake and ultimately reduce mortality rates in Lesotho.

## Abbreviations

ABM: Andersen Behavioral Model
DHS: Demographic and Health Survey
SDG: Sustainable Development Goals
SSA: Sub-Saharan Africa
LMIC: Low- and Middle-Income Countries
WHO: World Health Organizations.

## References

[1] Zhang S, Jin Z, Bao L, Shu P. The global burden of breast cancer in women from 1990 to 2030: assessment and projection based on the global burden of disease study 2019. Front Oncol. 2024;14:1364397. doi: 10.3389/fonc.2024.1364397 38966067 PMC11222408

[2] Magwesela FM, Msemakweli DO, Fearon D. Barriers and enablers of breast cancer screening among women in East Africa: a systematic review. BMC Public Health. 2023;23(1):1915. doi: 10.1186/s12889-023-16831-0 37794414 PMC10548570

[3] Afaya A, Japiong M, Konlan KD, Salia SM. Factors associated with awareness of breast cancer among women of reproductive age in Lesotho: a national population-based cross-sectional survey. BMC Public Health. 2023;23(1):621. doi: 10.1186/s12889-023-15443-y 37004021 PMC10067163

[4] Benitez Fuentes JD, Morgan E, de Luna Aguilar A, Mafra A, Shah R, Giusti F, et al. Global Stage Distribution of Breast Cancer at Diagnosis: A Systematic Review and Meta-Analysis. JAMA Oncol. 2024;10(1):71–8. doi: 10.1001/jamaoncol.2023.4837 37943547 PMC10636649

[5] Kim J, Harper A, McCormack V, Sung H, Houssami N, Morgan E, et al. Global patterns and trends in breast cancer incidence and mortality across 185 countries. Nat Med. 2025;31(4):1154–62. doi: 10.1038/s41591-025-03502-3 39994475

[6] Tolentino-Rodriguez L, Chkeir M, Pofagi V, Ahindu I, Toniolo J, Erazo A, et al. Breast cancer characteristics in low- and middle-income countries: An umbrella review. Cancer Epidemiol. 2025;96:102797. doi: 10.1016/j.canep.2025.102797 40081022

[7] Anyigba CA, Awandare GA, Paemka L. Breast cancer in sub-Saharan Africa: The current state and uncertain future. Exp Biol Med (Maywood). 2021;246(12):1377–87. doi: 10.1177/15353702211006047 33926257 PMC8243219

[8] Igbokwe KK. Comparative examination of breast cancer burden in sub-Saharan Africa, 1990–2019: estimates from Global Burden of Disease 2019 study. BMJ Open. 2024;14(3):e082492. 10.1136/bmjopen-2023-082492 PMC10982725 38553071

[9] Musekiwa A, Moyo M, Mohammed M, Matsena-Zingoni Z, Twabi HS, Batidzirai JM, et al. Mapping Evidence on the Burden of Breast, Cervical, and Prostate Cancers in Sub-Saharan Africa: A Scoping Review. Front Public Health. 2022;10:908302. doi: 10.3389/fpubh.2022.908302 35784211 PMC9246362

[10] Arnold M, Morgan E, Rumgay H, Mafra A, Singh D, Laversanne M, et al. Current and future burden of breast cancer: Global statistics for 2020 and 2040. Breast. 2022;66:15–23. doi: 10.1016/j.breast.2022.08.010 36084384 PMC9465273

[11] Vanderpuye V, Grover S, Hammad N, PoojaPrabhakar n, Simonds H, Olopade F. An update on the management of breast cancer in Africa. Infectious Agents and Cancer. 2017;12:1–12. 28228841 10.1186/s13027-017-0124-y PMC5307840

[12] Li N, Deng Y, Zhou L, Tian T, Yang S, Wu Y, et al. Global burden of breast cancer and attributable risk factors in 195 countries and territories, from 1990 to 2017: results from the Global Burden of Disease Study 2017. J Hematol Oncol. 2019;12(1):140. doi: 10.1186/s13045-019-0828-0 31864424 PMC6925497

[13] Azubuike SO, Muirhead C, Hayes L, McNally R. Rising global burden of breast cancer: the case of sub-Saharan Africa (with emphasis on Nigeria) and implications for regional development: a review. World J Surg Oncol. 2018;16(1):63. doi: 10.1186/s12957-018-1345-2 29566711 PMC5863808

[14] Mahumud RA, Gow J, Keramat SA, March S, Dunn J, Alam K, et al. Distribution and predictors associated with the use of breast cancer screening services among women in 14 low-resource countries. BMC Public Health. 2020;20(1):1467. doi: 10.1186/s12889-020-09557-w 32993596 PMC7526143

[15] Organization WH. WHO position paper on mammography screening: World Health Organization; 2014. 25642524

[16] Ba DM, Ssentongo P, Agbese E, Yang Y, Cisse R, Diakite B, et al. Prevalence and determinants of breast cancer screening in four sub-Saharan African countries: a population-based study. BMJ Open. 2020;10(10):e039464. doi: 10.1136/bmjopen-2020-039464 33046473 PMC7552834

[17] Anaba EA, Alor SK, Badzi CD, Mbuwir CB, Muki B, Afaya A. Drivers of breast cancer and cervical cancer screening among women of reproductive age: insights from the Ghana Demographic and Health Survey. BMC Cancer. 2024;24(1):920. doi: 10.1186/s12885-024-12697-6 39080553 PMC11290011

[18] Bamusi MT, Philip NE, Bhat LD. Women’s empowerment and its influence on the uptake of breast cancer screening in Tanzania: an analysis of 2022 Tanzania demographic health survey data. BMC Womens Health. 2024;24(1):495. doi: 10.1186/s12905-024-03345-z 39243087 PMC11378501

[19] Addo IY, Acquah E, Ayebeng C, Dickson KS. Influence of distance to health facilities on clinical breast cancer screening behaviour among women in five sub-Saharan African countries. BMC Public Health. 2023;23(1):915. doi: 10.1186/s12889-023-15782-w 37208657 PMC10199546

[20] Afaya A, Laari TT, Seidu AA, Afaya RA, Daniels-Donkor SS, Yakong VN, et al. Factors associated with the uptake of clinical breast examination among women of reproductive age in Lesotho: analysis of a national survey. BMC Cancer. 2023;23(1):114. doi: 10.1186/s12885-023-10566-2 36726101 PMC9890772

[21] Hailegebireal AH, Bizuayehu HM, Wolde BB, Tirore LL, Woldegeorgis BZ, Kassie GA, et al. The prevalence and predictors of clinical breast cancer screening in Sub-Saharan African countries: a multilevel analysis of Demographic Health Survey. Front Public Health. 2024;12:1409054. doi: 10.3389/fpubh.2024.1409054 39421823 PMC11483859

[22] Seyedkanani E, Hosseinzadeh M, Mirghafourvand M, Sheikhnezhad L. Breast cancer screening patterns and associated factors in Iranian women over 40 years. Scientific Reports. 2024;14(1):15274. 38961238 10.1038/s41598-024-66342-0 PMC11222508

[23] Ramathebane MM, Sooro MA, Kabuya RM, Sayed A-R. Knowledge and attitudes relating to cervical and breast cancer among women in Maseru, Lesotho. Afr J Prim Health Care Fam Med. 2022;14(1):e1–8. doi: 10.4102/phcfm.v14i1.3459 36546486 PMC9772699

[24] Lemma Seifu B, Mekuria Negussie Y, Abrham Asnake A, Daba Chinkey F, Melak Fente B, Alamrie Asmare Z. Determinants of breast cancer screening among women of reproductive age in sub-Saharan Africa: A multilevel analysis. PLoS One. 2024;19(12):e0312831. doi: 10.1371/journal.pone.0312831 39729464 PMC11676565

[25] Ramathebane M, Maja L, Sooro M, Sello M, Mokhethi M, Mputsoe K. Assessing challenges and opportunities of treating breast cancer in Lesotho. MRAJ. 2023;11(10). doi: 10.18103/mra.v11i10.4583

[26] Thabane K, Mashologu Y, Thabane L. Exploring factors associated with breast cancer screening among women aged 15-49 years in Lesotho. Pan Afr Med J. 2021;38:108. doi: 10.11604/pamj.2021.38.108.21110 33912278 PMC8051214

[27] Andersen RM. Revisiting the behavioral model and access to medical care: does it matter?. Journal of Health and Social Behavior. 1995;:1–10. 7738325

[28] Narcisse M-R, Shah SK, Hallgren E, Felix HC, Schootman M, McElfish PA. Factors associated with breast cancer screening services use among women in the United States: An application of the Andersen’s Behavioral Model of Health Services Use. Prev Med. 2023;173:107545. doi: 10.1016/j.ypmed.2023.107545 37201597 PMC10773561

[29] Akweh TY, Boakye BAJ, Adoku E, Teyko F, Tarkang EE. HIV testing uptake and its associated factors among Ghanaian men: insights from the 2022 Ghana demographic and health survey using the Anderson behavioral model. Discov Public Health. 2025;22(1). doi: 10.1186/s12982-025-00609-3

[30] Franck J-E, Ringa V, Cœuret-Pellicer M, Chauvin P, Menvielle G. The determinants of cervical cancer screening uptake in women with obesity: application of the Andersen’s behavioral model to the CONSTANCES survey. Cancer Causes Control. 2020;31(1):51–62. doi: 10.1007/s10552-019-01251-6 31797124

[31] Antabe R, Kansanga M, Sano Y, Kyeremeh E, Galaa Y. Utilization of breast cancer screening in Kenya: what are the determinants?. BMC Health Serv Res. 2020;20(1):228. doi: 10.1186/s12913-020-5073-2 32183801 PMC7079358

[32] Adegboye OA, Ezechukwu HC, Woodall H, Brough M, Robertson-Smith J, Paba R, et al. Media Exposure, Behavioural Risk Factors and HIV Testing among Women of Reproductive Age in Papua New Guinea: A Cross-Sectional Study. Trop Med Infect Dis. 2022;7(2):30. doi: 10.3390/tropicalmed7020030 35202225 PMC8875656

[33] Bazyar M, Kakaei H, Azadi H, Jalilian M, Mansournia MA, Malekan K, et al. Self-rated health status and associated factors in Ilam, west of Iran: results of a population-based cross-sectional study. Front Public Health. 2025;12:1435687. doi: 10.3389/fpubh.2024.1435687 39839384 PMC11747038

[34] Rahman A, Tohan MM, Islam A, Saha BR, Kundu S. Prevalence and social determinants of self-reported health status among reproductive age women in Nepal. Archives of Women’s Mental Health. 2024;:1–11. 10.1007/s00737-024-01528-z 39560776

[35] K. Van Ginneken J, Groenewold G. A Single- vs. Multi-Item Self-Rated Health Status Measure: A 21-Country Study. TOPHJ. 2012;5(1):1–9. doi: 10.2174/1874944501205010001

[36] Whittaker TA, Furlow CF. The Comparison of Model Selection Criteria When Selecting Among Competing Hierarchical Linear Models. J Mod App Stat Meth. 2009;8(1):173–93. doi: 10.22237/jmasm/1241136840

[37] Ayebeng C, Okyere J, Okantey C, Addo IY. Multifaceted barriers associated with clinical breast examination in sub-Saharan Africa: A multilevel analytical approach. PLoS One. 2025;20(1):e0316800. doi: 10.1371/journal.pone.0316800 39804868 PMC11730425

[38] Lamichhane B, Adhikari B, Poudel L, Pandey AR, Kakchhapati S, K C SP, et al. Factors associated with uptake of breast and cervical cancer screening among Nepalese women: Evidence from Nepal Demographic and Health Survey 2022. PLOS Glob Public Health. 2024;4(3):e0002971. doi: 10.1371/journal.pgph.0002971 38466682 PMC10927089

[39] Abebe Gebreegziabher Z, Semagn BE, Walle AD, Belay MA, Wondie WT, Degefaw GD, et al. Clinical breast examination and its associated factors among reproductive age women in Ghana: multilevel logistic regression analysis. Front Oncol. 2024;14:1413076. doi: 10.3389/fonc.2024.1413076 39777338 PMC11703916

[40] Antabe R, Sano Y, Amoak D, Kyeremeh E. Breast cancer screening among married women in Tanzania: does household structure matter?. Discover Social Science and Health. 2025;5(1):1–11.

[41] Mahumud RA, Alam K, Keramat SA, Renzaho AMN, Hossain MG, Haque R, et al. Wealth stratified inequalities in service utilisation of breast cancer screening across the geographical regions: a pooled decomposition analysis. Arch Public Health. 2020;78:32. doi: 10.1186/s13690-020-00410-5 32528677 PMC7285540

[42] Ebrahimoghli R, Aghaei MH, Azami-Aghdash S, Houssami N. Uptake of breast cancer screening practices in low- and middle-income countries: a systematic review and meta-analysis. J Natl Cancer Inst. 2025;117(1):29–39. doi: 10.1093/jnci/djae187 39133184

[43] Mukem S, Sriplung H, McNeil E, Tangcharoensathien V. Breast cancer screening among women in Thailand: analyses of population-based household surveys. J Med Assoc Thai. 2014;97(11):1106–18. 25675674

[44] Ngan TT, Jenkins C, Minh HV, Donnelly M, O’Neill C. Breast cancer screening practices among Vietnamese women and factors associated with clinical breast examination uptake. PLoS One. 2022;17(5):e0269228. doi: 10.1371/journal.pone.0269228 35622840 PMC9140272

[45] Alkhawaldeh A, ALBashtawy M, Rayan A, Abdalrahim A, Musa A, Eshah N, et al. Application and Use of Andersen’s Behavioral Model as Theoretical Framework: A Systematic Literature Review from 2012-2021. Iran J Public Health. 2023;52(7):1346–54. doi: 10.18502/ijph.v52i7.13236 37593505 PMC10430393

[46] Kangmennaang J, Mkandawire P, Luginaah I. Breast cancer screening among women in Namibia: explaining the effect of health insurance coverage and access to information on screening behaviours. Glob Health Promot. 2019;26(3):50–61. doi: 10.1177/1757975917727017 28944716

